# Evaluation of the C-reactive Protein/Albumin Ratio and Fluid Overload in Critically Ill Children and Adolescents Aged One Month to 18 Years

**DOI:** 10.7759/cureus.75252

**Published:** 2024-12-06

**Authors:** Om S Chaurasiya, Sachin Rana, Kawalpreet Chhabra, Mayank Singh

**Affiliations:** 1 Paediatrics, Maharani Laxmi Bai Medical College, Jhansi, IND

**Keywords:** crp-albumin ratio, fluid overload, mortality predictors, prism iii score, prognostic factor, triage

## Abstract

Introduction

The pediatric intensive care unit (PICU) is a specialized area for treating critically ill infants and children. However, some of these children may experience poor outcomes, including death. However, it is necessary to predict the prognosis for critically ill patients as early as possible to commence triage as well as an early and effective intervention to prevent mortality. The objective was to evaluate C-reactive protein (CRP) to albumin ratio and fluid overload as predictors of mortality in critically ill children and adolescents (one month to 18 years).

Methodology

This was a prospective, observational study conducted on 100 critically ill cases admitted to the PICU in a tertiary care hospital. Demographic profiles and clinical manifestations were noted, and baseline investigations were carried out. Children were provided treatment according to PICU protocols. CRP and serum albumin levels were measured, and fluid intake and output were documented in detail.

Results

The mean CRP/albumin ratio in the survivor group was 16.39±14.161, while in the non-survivor group, it was reported to be 12.95±11.905. The mean CRP/albumin ratio among the patients who required ventilation for <3 days, 3-5 days, and >5 days were 16.91±14.35, 12.90±10.80, and 2.0±0.321, respectively. The mean value for fluid overload in the survivor group was 10.90±7.44 and in the non-survivor group was 20.54±18.727. The mean fluid overload among the patients who required ventilation for <3 days, 3-5 days, and >5 days were 14.61±9.639, 19.30±13.21, and 20.74±19.81, respectively. CRP/albumin ratio was directly related to increased ventilation in critically ill patients, inversely proportional to ventilatory stay. Fluid overload was directly associated with the development of multiple organ dysfunction syndrome (MODS).

Conclusion

It was concluded that fluid overload can be used as a predictor of poor outcome though CRP/albumin ratio still warrants further studies.

## Introduction

Critically ill children require special attention, and after triage in an emergency, they are transferred to a specialized area within a hospital in the pediatric intensive care unit (PICU) for intensive monitoring. In PICU, most of the children have critical illness, and sometimes, these children may end up with poor outcomes, resulting in death. Prognostic scores help predict the disease condition's outcome, anticipate deterioration and improvement as early as possible, and prevent mortality. Sepsis, specifically severe sepsis, and septic shock remains a major cause of morbidity and mortality worldwide [1]. The mortality rate of severe sepsis is 20-30%, accounting for about 30-50% of hospital deaths [2,3].

Many studies have evaluated parameters or biomarkers used for prognosticating critically ill patients. A simple, quick, and accessible parameter is needed to confirm treatment response and anticipate mortality in ICU patients. In critically ill patients, high CRP levels have been linked to death and prognosis [4,5]. Conversely, low blood albumin levels are linked to a worse prognosis and higher death rates [6,7]. When combined, CRP and albumin can provide information on inflammation and nutrition, making them more useful prognostic markers for outcomes in various disorders [8,9]. A common issue seen in PICUs is fluid imbalances and hemodynamic instability. Aggressive fluid administration can lead to fluid overload, a condition in which there is a positive fluid balance in the patient. Fluid overload itself increases the risk of morbidity and mortality in a population who are already at risk of adverse effects of fluid overload. Though many prognostic scoring systems are available for critically ill patients, these are complex or require expensive investigations, precluding their application, especially in resource-limited settings [10-12]. We aimed to evaluate CRP/albumin ratio and fluid overload as predictors of outcomes in critically ill pediatric patients.

## Materials and methods

Study design and participants

This prospective, observational study enrolled 100 cases admitted to the PICU in the Department of Pediatrics at Maharani Laxmi Bai Medical College, Jhansi, over a 13-month period from 1st April 2023 to 30th April 2024. The study was undertaken after approval from the institution's ethical committee (Institutional Ethics Committee of Maharani Laxmi Bai Medical College, Jhansi, issued approval for IEC/I/2022-2023 dated 03/03/2023). Consent was obtained or waived by all participants in this study.

Selection Criteria

All critically ill children between the ages of one month and less than 18 years admitted to the PICU were included after obtaining informed consent from the parents. Children with congenital heart disease, nephrotic syndrome, chronic liver disease, or any other condition causing hypoalbuminemia (such as severe acute malnutrition, protein-losing enteropathy, or malabsorption syndrome), and those who did not provide consent were excluded from the study. Enrolled cases underwent a comprehensive assessment that included detailed history-taking, including chronic illnesses and long-term medication use, followed by a thorough general and systemic examination, anthropometric measurements, and special investigations or interventions as clinically indicated.

Data sources and variables

Blood Collection and Analysis

Approximately 4 mL of blood was collected on the day of admission via venipuncture in serum-separating vacutainers (Greiner Bio-One Vacuette, Austria). Blood was allowed to clot for 30 minutes at room temperature before centrifugation at 3,000 rpm for 10 minutes to separate serum. Serum C-reactive protein (CRP) levels were measured using a high-sensitivity turbidometric immunoassay (Beckman Coulter AU5800, reagent kit: Beckman Coulter CRP Reagent, Brea, USA). Serum albumin levels were measured using an automated analyzer with the bromocresol green dye-binding method (Roche Cobas C311, reagent kit: Roche Diagnostics Albumin Assay, Mannheim, Germany). The CRP/albumin ratio was calculated by dividing serum CRP levels (mg/L) by serum albumin levels (g/dL).

Fluid Balance Monitoring

Fluid intake and output were meticulously documented. Fluid intake included all forms of enteral and parenteral intake, such as fluid with medication, intravenous maintenance fluids, all forms of nutritional support, and blood products. Fluid output included urine, stool, bleeding from any site, drainage from tubes inserted into body cavities, and aspirated fluids from procedures like pleural or ascitic tapping. The degree of fluid overload was calculated using the formula: Fluid Overload = [(Fluid intake- Fluid output)/body weight at PICU admission] × 100.

Fluid overload was defined as a fluid accumulation of ≥25%. Treatment protocols in the PICU included ventilatory support, either non-invasive or invasive, inotropic support with agents such as dopamine, dobutamine, and norepinephrine, and empirical antibiotic therapy adjusted based on microbiological culture results. Management adhered to the Indian Society of Critical Care Medicine (ISCCM) Pediatric Guidelines to ensure consistency in care delivery. The outcome for each child was documented as survival without organ dysfunction, survival with multi-organ dysfunction syndrome, or death.

Statistical analysis

The study involved the statistical analysis of 100 critically ill pediatric patients admitted to the PICU. Statistical analyses were performed using IBM SPSS Statistics Version 26.0 (IBM Corp., Armonk, NY, USA). Continuous variables were summarized as mean ± standard deviation and compared between survivor and non-survivor groups using independent t-tests for normally distributed data, or Mann-Whitney U tests for non-normally distributed data. Categorical variables were expressed as frequencies and percentages. A p-value of <0.05 was considered statistically significant.

## Results

The mean age of the sample population was 87.78±67.588 months. The mean age in the survivor group was 95.34±69.23 months and that for patients who died was 73.08±62.66 months. The male-female ratio was 0.92:1, whereas among survivors, it was 0.78:1, and in patients who expired was 1.2:1. The mean weight, height, and BMI of the patient in the survivor and non-survivor groups was 22.74±14.392 and 19.12±13.137, 114.20±14.392 and 104.02±36.823, 15.65±3.946 and 15.43±3.561, respectively. There was no significant difference between the survivor and non-survivor groups in weight, height, and BMI.

The majority of the cases, 15 (15%) were respiratory illness, followed by dengue (12%), 11% of tuberculosis, 10% of poisoning, meningitis/meningoencephalitis (9%), seizure disorder (6%), 8% of acute gastroenteritis with severe dehydration with shock, septic shock, and DKA comprised of 4% each, acute hepatic encephalopathy 2%, UTI 2%, pancytopenia 2%, diphtheria 2%, malaria 1%, and other miscellaneous group of disease, which comprised of 11% that includes aspiration pneumonitis, tetanus, von-Willebrand disease, Guillain-Barre syndrome, brain stem glioma, liver abscess, west syndrome, and acute lymphoid leukemia. No significant difference was found between the CRP/albumin ratio among patients in the survivor and non-survivor groups. The mean CRP/albumin ratio of the patient was 15.23±0.755. The mean level in the survivor group was 16.39±14.161, and among non-survivors was reported to be 12.95±11.905, as shown in Table 1. 

**Table 1:** Distribution according to CRP/serum albumin ratio and fluid overload A t-test was used to determine the p-value. A p-value less than 0.05 was considered significant. The calculated t-score for the CRP/serum albumin ratio between survivors and non-survivors is 2.09. CRP, C-reactive protein

Variables	Survivor	Non-survivor	p-value
CRP/serum albumin ratio			0.2284
Range	1.3-83.2	0.4-40.96	
Median	14.52	8.56	
Mean ± SD	16.39±14.161	12.95±11.905	
Fluid overload (%)			
Range	4-38	5-62.5	
Median	12.05	14	

There was a significant difference in fluid overload between the patients of the survivor (10.90±7.44) and non-survivor groups (20.64±18.72). This result indicated that mortality was more likely in patients with fluid overload than in patients without fluid overload. CRP/albumin ratio was non-significant in the patient with respect to ionotropic use, with the mean CRP/albumin ratio of the patients who did not require inotropes, and those who required was 16.12±11.17 and 14.67±14.66, respectively. Patients with more fluid overload had increased requirement of ionotropic support with mean 13.63±6.82 (not required inotropes) and 17.85±13.08 (required inotropes). The same has been depicted in Table 2. 

**Table 2:** Comparison between the CRP/albumin ratio and fluid overload to need of inotropes A t-test was used to determine the p-values. A p-value less than 0.05 was considered significant. The calculated t-scores for the CRP/albumin ratio and fluid overload comparisons with inotrope usage are 0.88 and 0.69, respectively. CRP, C-reactive protein

Variables	Inotropes (n=62)	No inotropes (n=38)	P-value
CRP/albumin			
Mean ± SD	14.67±14.66	16.12±11.17	0.6029 (NS)
Fluid overload			
Mean ± SD	17.85±13.08	13.63±6.82	0.0689 (NS)

A higher CRP/albumin ratio was related to a shorter duration of ventilation along with a longer duration of PICU stay. Patients with more fluid overload had shorter duration of stay, as shown in Table 3. No significant difference was found between the CRP/albumin ratio and patients who developed multiple organ dysfunction syndrome (MODS). Patients with more fluid overload had more chances to develop MODS, as shown in Table 4. The fluid overload and CRP/albumin ratio among the patients who developed MODS and those who did not develop MODS was 38.64±19.625 and 14.36±9.54, 13.16±9.80 and 15.43±13.73, respectively.

**Table 3:** Comparison of CRP/albumin ratio and fluid overload to duration of ventilation and hospital stay CRP, C-reactive protein

Variables	CRP/albumin (mean ± SD)	Fluid overload (mean ± SD)
Ventilation duration (days)		
<3 (n=66)	16.91±14.35	14.61±9.639
3-5 (n=31)	12.90±10.80	19.30±13.21
>5 (n=3)	2.0±0.321	20.74±19.81
Hospital stay (days)		
0-3 (n=8)	12.13±8.356	29.53±22.28
4-7 (n=38)	14.72±12.034	17.43±9.51
>7 (n=54)	16.04±14.92	13.44±8.55

**Table 4:** Comparison of fluid overload and CRP/albumin ratio with MODS A t-test was used to determine the p-values. A p-value less than 0.05 was considered significant. The calculated t-scores for the comparisons of fluid overload and CRP/albumin ratio with MODS are 5.86 and -0.75, respectively. CRP, C-reactive protein; MODS, multiple organ dysfunction syndrome

Variables	MODS		P-value
	Yes (n=9)	No (n=91)	
Fluid overload (mean ± SD)	38.64±19.625	14.36±9.54	<0.0001
CRP/albumin ratio (mean ± SD)	13.16±9.80	15.43±13.73	0.6302

## Discussion

Mortality and morbidity in the limited number of PICUs in our country are significant concerns due to the disproportionate ratio of available PICU facilities to the number of patients requiring critical care. This highlights the urgent need for a simple, effective predictive scoring system to aid in resource allocation and patient management. In this study, the CRP/albumin ratio was explored as a potential predictor of outcomes in critically ill pediatric patients. While CRP alone has been shown to have limited predictive value for mortality in the pediatric population, combining it with albumin levels offers a more robust indicator [13]. Hypoalbuminemia, in particular, is recognized as a reliable, cost-effective predictor of outcomes in both adults and children under critical care conditions [13]. Fluid balance has also been identified as a significant independent predictive factor for mortality in critically ill patients, further supporting the importance of evaluating these parameters in outcome prediction [14,15].

There still is a lacuna with regard to the establishment of these markers as predictors of morbidity, especially in resource-limiting areas such as ours. Thus, this study was conducted as a modest attempt to fill some part of the gap. The association of mortality in critically ill children has also been established with CRP to albumin ratio and fluid balance [16,17]. In our study, the mean age was 87.78±67.588 months, with a range of 1.5-204 months. Among the patients who survived, the mean age was 95.34±69.23 months, and that for patients who expired was 73.08±62.66. The male-female ratio was 0.92:1, whereas among survivors, it was 0.78:1, and in patients who expired was 1.2:1. A different demographic profile of the study population was observed in studies by Shereen A. Mohamed, where the median age was 11 months with male and female ratio of 1.2:1 [18]. In contrast, it was 32+29.3 months with a male-to-female ratio of 1.3:1 in the study done by Hina Aktar et al. [19]. This might be because of the late presentation of female patients or some other social reason that would require in-depth probing.

The average albumin levels reported in our study were 3.61±0.496 g/dL. The mean albumin levels in the non-survivor group were 3.59±0.405 g/dL, while in survivors, it was 3.61±0.54 g/dL. No significant difference between the serum albumin level and CRP/albumin ratio in survivor and non-survivor group patients was observed. Horowitz and Tai established the incidence of hypoalbuminemia in critically ill children [20]. They reported albumin levels of 1.64±0.46 g/dL in their patient group. Low serum albumin has also been predicted as a predictor of mortality in the PICU by Kim Y and the team [21]. Tiwari L et al. reported mean albumin levels of 2.61 (SD: 0.67) g/dL in the population and mean albumin levels of 2.00 (SD: 0.33) g/dL in the hypoalbuminemia group, whereas 3.1 g/dL (SD: 0.45) in the normal-albumin emic group [22]. In patients receiving inotrope support, the median CRP/albumin ratio was significantly greater (11.70 and 3.68, respectively). Furthermore, Shreen M. A. et al. found that serum albumin was considerably lower among patients in need of inotropes, and the median CRP was much higher [18]. According to Hina Akhtar et al., the median CRP/albumin ratio in non-survivors was significantly greater than that in survivors (18.60 versus 4.65, respectively) [19].

Serum albumin and CRP levels also showed a substantial difference between survivors and non-survivors. There was a significant difference in fluid overload between the patients of the survivor (10.90±7.44) and non-survivor groups (20.64±18.72). This result indicates that mortality was more likely in patients with fluid overload than in patients without fluid overload. Similar results have been observed in other studies. Ida Bagus Ramajaya Sutawan showed that the mean cumulative fluid accumulation percentage was significantly higher in the non-survivor group (7.9±12.9%) compared with the survivor group (1.4±8.2%) [23].

Patients with more fluid overload had an increased requirement for ionotropic support and the need for and duration of ventilation in our study. However, it was inversely related to the duration of hospital stay. Thus it was demonstrated that increased mortality is associated with increased fluid overload. Fluid overload is directly associated with the development of MODS and mortality. It has a significant correlation with the increased requirement of ionotropic support, the demand for ventilation, and ventilatory stay but is inversely related to the duration of PICU stay. CRP/albumin ratio within 24 hours of admission to a PICU was not found to be a reliable predictor of mortality in the pediatric age group. CRP/albumin ratio was directly related to increased ventilation in critically ill patients, inversely proportional to ventilatory stay, and directly proportional to longer duration of PICU stay, but no significant difference was found between the CRP/albumin ratio and need for inotropes.

Strengths

This study has several notable strengths. It employed a prospective design, which allowed for systematic data collection and minimized recall bias. The study provides valuable insights into the CRP/albumin ratio and fluid overload as predictors of outcomes in critically ill children in a resource-limited setting, where such data are often scarce. Additionally, the findings have potential applicability for guiding the development of predictive tools in similar low-resource settings. The focus on fluid balance and the CRP/albumin ratio as simple and cost-effective markers can inform future research and clinical practice in pediatric critical care.

Limitations

This observational study was conducted at a single center, which limits the generalizability of the findings. Furthermore, daily weight measurements were not recorded due to the severity of illness in many cases, which prevented a more objective daily assessment of positive fluid balance. As both the CRP/albumin ratio and fluid overload were parameters of interest in the study, the number of exclusion criteria was higher, reducing the generalizability to children with conditions like congenital heart disease or nephrotic syndrome. This limitation underscores the need to develop additional prognostic markers for critically ill children who meet exclusion criteria for studies like this.

## Conclusions

This study highlights the utility of fluid overload as a potential predictor of poor outcomes in critically ill children and adolescents. Fluid overload was positively correlated with the development of multi-organ dysfunction syndrome (MODS) and was found to significantly influence outcomes, supporting its role as a parameter for early risk stratification and intervention in the pediatric intensive care setting. In contrast, while a high CRP/albumin ratio was associated with increased ventilation requirements and longer mechanical ventilation durations, the study did not find a statistically significant difference in CRP/albumin ratios between survivors and non-survivors. This suggests that while CRP/albumin ratio may provide some clinical insights, further research is required to establish its predictive value in pediatric critical care. Assessment of these parameters early during PICU admission may assist clinicians in risk stratification and timely interventions to improve prognostic accuracy and patient care. However, further multicenter studies with larger sample sizes are warranted to confirm these findings and validate fluid overload as a reliable predictor of mortality in this population.
